# microRNA-335 inhibits proliferation, cell-cycle progression, colony formation, and invasion via targeting *PAX6* in breast cancer cells

**DOI:** 10.3892/mmr.2021.12278

**Published:** 2021-07-09

**Authors:** Yuanbiao Meng, Quanqing Zou, Tianqi Liu, Xiaoyong Cai, Yubin Huang, Jinfei Pan

Mol Med Rep 11: 379-385, 2015; DOI: 10.3892/mmr.2014.2684

Following the publication of this paper, it was drawn to the Editors' attention by a concerned reader that the cell cycle assay data shown in Fig. 4A, and the western blotting assay data shown in Fig. 4B, were strikingly similar to data appearing in different form in other articles by different authors; furthermore, there were other possible anomalies associated with these data. Owing to the fact that the contentious data in the above article had already been published elsewhere, or were already under consideration for publication, prior to its submission to *Molecular Medicine Reports*, the Editor has decided that this paper should be retracted from the Journal. The authors were asked for an explanation to account for these concerns, but the Editorial Office did not receive any reply. The Editor apologizes to the readership for any inconvenience caused.

